# Tonic NMDAR Currents of NR2A-Containing NMDARs Represent Altered Ambient Glutamate Concentration in the Supraoptic Nucleus

**DOI:** 10.1523/ENEURO.0279-23.2023

**Published:** 2024-02-09

**Authors:** Hyunjin Shin, Ramesh Sharma, Chiranjivi Neupane, Thuy Linh Pham, Su Eun Park, So Yeong Lee, Hyun-Woo Kim, Young Min Bae, Javier E. Stern, Jin Bong Park

**Affiliations:** ^1^Department of Physiology & Medical Science, College of Medicine & Brain Research Institute, Chungnam National University, Daejeon 35015, South Korea; ^2^Laboratory of Veterinary Pharmacology, College of Veterinary Medicine and Research Institute for Veterinary Science, Seoul National University, Seoul 08826, Republic of Korea; ^3^Department of Physiology, Konkuk University School of Medicine, Chungju 27478, Republic of Korea; ^4^Neuroscience Institute and Center for Neuroinflammation and Cardiometabolic Diseases, Georgia State University, Atlanta, Georgia 30302

**Keywords:** NMDARS, NR2A, SON MNCs

## Abstract

NMDA receptors (NMDARs) modulate glutamatergic excitatory tone in the brain via two complementary modalities: a phasic excitatory postsynaptic current and a tonic extrasynaptic modality. Here, we demonstrated that the tonic NMDAR-current (*I*_NMDA_) mediated by NR2A-containing NMDARs is an efficient biosensor detecting the altered ambient glutamate level in the supraoptic nucleus (SON). *I*_NMDA_ of magnocellular neurosecretory cells (MNCs) measured by nonselective NMDARs antagonist, AP5, at holding potential (*V*_holding_) −70 mV in low concentration of ECF Mg^2+^ ([Mg^2+^]_o_) was transiently but significantly increased 1-week post induction of a DOCA salt hypertensive model rat which was compatible with that induced by a NR2A-selective antagonist, PEAQX (*I*_PEAQX_) in both DOCA-H_2_O and DOCA-salt groups. In agreement, NR2B antagonist, ifenprodil, or NR2C/D antagonist, PPDA, did not affect the holding current (*I*_holding_) at *V*_holding_ −70 mV. Increased ambient glutamate by exogenous glutamate (10 mM) or excitatory amino acid transporters (EAATs) antagonist (TBOA, 50 mM) abolished the *I*_PEAQX_ difference between two groups, suggesting that attenuated EAATs activity increased ambient glutamate concentration, leading to the larger *I*_PEAQX_ in DOCA-salt rats. In contrast, only ifenprodil but not PEAQX and PPDA uncovered *I*_NMDA_ at *V*_holding_ +40 mV under 1.2 mM [Mg^2+^]_o_ condition. *I*_ifenprodil_ was not different in DOCA-H_2_O and DOCA-salt groups. Finally, NR2A, NR2B, and NR2D protein expression were not different in the SON of the two groups. Taken together, NR2A-containing NMDARs efficiently detected the increased ambient glutamate concentration in the SON of DOCA-salt hypertensive rats due to attenuated EAATs activity.

## Significance Statement

The NMDAR-mediated excitatory tone between cells is transmitted via phasic activation of synaptic NMDARs (EPSCs) and tonic activation of extrasynaptic NMDARs (*I*_NMDA_) in the brain. The activation of NMDARs depends on the glutamate concentration, NMDAR subunit composition, and their subcellular localization, as well as the membrane potential. Therefore, the mechanism of NMDAR-mediated excitatory tone varies in different pathophysiological conditions. Our results show that the *I*_NMDA_ in nondepolarized and depolarized neurons is dominantly mediated by NR2A- and NR2B-containing NMDARs, respectively, and the former efficiently detects the ambient glutamate concentration in the supraoptic magnocellular neuroendocrine cells of normal and hypertensive rats. This study shows that NR2A-containing NMDARs could be a biosensor detecting ambient glutamate concentration in the brain.

## Introduction

Glutamate is a major excitatory neurotransmitter in the supraoptic nucleus (SON; van den Pol et al., 1990), which is composed of vasopressin and oxytocin magnocellular neurosecretory cells (MNCs). These neurons are known to play critical roles in fluid balance and cardiovascular and reproductive homeostasis (Silverman and Zimmerman, 1983). Neurohumoral activation has a direct impact on morbidity/mortality in cardiovascular diseases (Cohn et al., 1984; Yemane et al., 2010). In addition to the classical transient excitatory postsynaptic currents (EPSCs) mediated by synaptic receptors, glutamate generates a tonic, sustained excitatory current (*I*_NMDA_) when it binds to extrasynaptic NMDARs (eNMDARs) that strongly stimulate firing activity in SON MNCs (Fleming et al., 2011). NMDARs are heterotetramers composed of two NR1 subunits and two NR2 subunits. NMDARs containing NR2A, B, C, and D subunits encoded by four different genes (*GluN2A-D*) exhibit distinct electrophysiological and pharmacological properties as well as different subsynaptic distributions and expression profiles. For example, eNMDARs containing NR2B or NR2D subunit could mediate *I*_NMDA_ with their tonic activation (Fleming et al., 2011; Neupane et al., 2021), while NR2A-containing NMDARs, predominantly found in synaptic space, mediate EPSCs. NR2D-containing eNMDARs even generate a “Mg^2+^-resistant” *I*_NMDA_ activated in nondepolarized SON MNCs under the physiological concentration of [Mg^2+^]_o_ (Neupane et al., 2021). Given that elevated glutamatergic excitatory tone supports exacerbated activity of MNCs (Biancardi et al., 2010; Li et al., 2014; Glass et al., 2015; Zhang et al., 2017), which in turn contributes to neurohumoral activation during cardiovascular diseases, elucidating the precise roles of different NMDARs and their subunit plasticity altering MNCs activity in diseases such as hypertension and heart failure is of critical importance.

Under conditions requiring strong secretion of neurohypophysial hormones, there is a pronounced reduction in the astrocytic coverage of SON NMCs (Theodosis and Poulain, 1993; Bobak and Salm, 1996). Neuroglia remodeling in response to physiological challenges resulted in blunted glutamate transporter (GLT) activity leading to increased ambient glutamate in the SON (Fleming et al., 2011), which may depolarize the neurons despite the Mg^2+^ block of NMDARs (Mayer et al., 1984; Nowak et al., 1984). Given that robust NR2D protein expression in the SON MNCs is uncommon in the adult brain (Doherty and Sladek, 2011) and that NR2D generates an “Mg^2+^-resistant” *I*_NMDA_ of MNCs in a state-dependent manner (Neupane et al., 2021), we investigated whether NR2D-containing eNMDARs contribute to exacerbated *I*_NMDA_ in SON neurons during hypertension and whether this finding could be a mechanism contributing in turn to neurohumoral activation in this cardiovascular disease. Unexpectedly, our results demonstrate that in DOCA-salt hypertensive rats, a decreased activity of excitatory amino acid transporters (EAATs) resulted in increased ambient glutamate levels to potentiate a Mg^2+^-sensitive *I*_NMDA_ mediated by NMDAR-containing NR2A rather than NR2B and NR2D.

## Materials and Methods

### DOCA-salt hypertension model

All animal experimentation was approved by the Institutional Animal Care and Use Committee and was conducted in accordance with the National Institutes of Health Guide for the Care and Use of Laboratory Animals. Male 5-week-old Sprague Dawley (SD) rats weighing 120–180 g were purchased from Animals. All animals were housed on a 12-hour light/dark cycle and had access to food ad libitum throughout the experiments. After 1 week of acclimatization, rats were anesthetized with avertin (250 mg/kg, i.p.; Millipore Sigma) and underwent unilateral nephrectomy (left kidney) as previously described (Prahalathan et al., 2012). Briefly, a lateral abdominal incision was made to access the left kidney for its resection and sutured after the removal of the kidney. After 1 week, DOCA (D7000-5G, Sigma-Aldrich) was implanted subcutaneously in all nephrectomized rats, and animals were randomly assigned to either the H_2_O group (DOCA-H_2_O) or salt group (DOCA-salt). The DOCA-salt group had their water replaced with a mixture of 0.8% NaCl and 0.2% KCl in tap water to drink until killed (after 1 or 4 weeks).

### Blood pressure measurement

Systolic blood pressure was measured once a week for a month by tail cuff method as previously described (Kubota et al., 2006). Rats were restrained in a cylindrical restrainer at 37°C for 30 min to acclimatize them to the apparatus (noninvasive blood pressure system, CODA; Kent Scientific Corporation) before blood pressure recordings were made. Systolic blood pressure was measured in awake rats using a noninvasive tail cuff blood pressure measuring system (PowerLab/8SP data acquisition system, ADInstruments) before DOCA treatment and on the 1, 2, 3, and 4 weeks of DOCA treatment. The physiological data were analyzed using the LabChart 6.1 Pro software (ADInstruments). Averaged blood pressure from at least five consecutive readings obtained from each rat was recorded as final blood pressure.

### Water intake, urine output, serum osmolality, and urine osmolality measurements

After 6th day of DOCA treatment, both DOCA-H20 and DOCA-salt groups were individually housed in metabolic cages provided with H_2_O or salt for 24 hours before sample collection. Urine output and water intake were measured within the following 24 hours. In addition, on the 7th day, a fresh urine sample was collected in a tube, and animals were anesthetized with avertin (250 mg/kg, i.p.; Millipore Sigma) for blood collection by cardiac puncture. Both urine and blood were centrifuged. After centrifugation, urine and serum osmolality were measured using the freezing point depression method and a micro-osmometer (model 210, Fiske Associates).

### Electrophysiology and data analysis

Patch-clamp recordings from SON MNCs were obtained from acutely prepared hypothalamic slices (300 µm) as previously described (Neupane et al., 2021). Briefly, rats were decapitated under avertin anesthesia (Avertin, 200 mg/kg, i.p.), and the brains were quickly extracted. Sectioned slices were incubated in artificial CSF (aCSF) containing (in mM) 126 NaCl, 5 KCl, 1.2 MgCl_2_, 26 NaHCO_3_, 1.2 NaH_2_PO_4_, 10 glucose, and 2.4 CaCl_2_, pH 7.3–7.4, and saturated with 95% O_2_ and 5% CO_2_ within the slice holder for 1 h at 34°C in the presence of 3 µM glutamic acid. Single hemisectioned slices were transferred to a recording chamber perfused with aCSF saturated with 95% O_2_ and 5% CO_2_ and maintained at 34°C. All electrophysiological measurements were recorded using a MultiClamp 700B (Molecular Devices). The current output was filtered at 1 kHz and digitized at 10 kHz (Digidata 1440 and pClamp 10.2 software, Molecular Devices). Data were excluded if the series resistance was not stable throughout the entire recording (20% change) or if neuronal input resistance (IR) was <550 MΩ at the beginning of the recording. The NMDA receptor-mediated tonic current (tonic *I*_NMDA_) was defined as changes in the holding current (*I*_holding_) in the presence of ionotropic GABA receptor antagonists and was calculated by the difference in *I*_holding_ measured as the average of a 2-minute steady-state baseline segment obtained before and after the application of NMDAR antagonists. *I*_NMDA_ was recorded and calculated at −70 mV with low [Mg^2+^]_o_ (20 µM) or +40 mV with normal aCSF containing 1.2 mM [Mg^2+^]_o_ unless otherwise stated. Event detection and analysis of spontaneous EPSCs were carried out using MiniAnalysis software (Synaptosoft) at *V*_holding_ −70 mV, as previously described (Park et al., 2007). The detection threshold was set at −20 pA and 75 pA/ms for EPSC amplitude and area, respectively. From extracted EPSCs, frequency, amplitude, and decay time constant were calculated. EPSC decay time constants were calculated from single exponential fits.

Drugs were added to the aCSF perfusing solution at differing concentrations. The final concentration of dimethylsulfoxide (DMSO) was <0.05%, when used as a vehicle. DL-2-amino-5-phospho-nopentanoic acid (AP5), (2S*, 3R*) -1-(phenanthren-2-carbonyl) piperazine-2, 3-dicarboxylic acid (PPDA), [[[(1*S*)-1-(4-bromophenyl)ethyl]amino](1,2,3,4-tetrahydro-2,3-dioxo-5-quinoxalinyl)methyl] phosphonic acid tetrasodium salt (PEAQX) and DL-*threo*-β-benzyloxyaspartic acid (TBOA) were all purchased from Tocris Bioscience. All other drugs were purchased from Sigma-Aldrich.

### Western blotting

Brain tissue punches containing the SON were collected from 300 µM coronal hypothalamic slices (three sequential slices/rats) from each brain as described previously (Potapenko et al., 2012). Protein was extracted from the SON punches using a mixture of a protease inhibitor and radioimmunoprecipitation assay (RIPA) lysis buffer and quantified using a bicinchoninic acid (BCA) assay kit (Thermo Fisher Scientific). Approximately 50 µg of protein was separated on a 10% sodium dodecyl sulfate (SDS)-polyacrylamide gel and transferred to a nitrocellulose membrane. The membranes were then blocked with TBST (0.1% Tween 20 in 1× Tris-buffered saline) containing 5% skimmed milk for 1 h at room temperature. The blot was then probed with primary antibodies against NMDAR NR2A (1:1,000; catalog #AGC-002, Alomone Labs; RRID: AB_2756596), NR2B (1:1,000; catalog #06-600, Millipore; RRID: AB_310193), and NR2D subunits (1:1,000; catalog #AGC-020, Alomone Labs; RRID: AB_10658334) overnight at 4°C. Next, the membranes were exposed to horseradish peroxidase-conjugated goat anti-rabbit (catalog #7074, Cell Signaling Technology; RRID: AB_2099233) and anti-mouse (catalog #31430, Thermo Fisher Scientific; RRID: AB_228307) secondary antibody (1:1,000) at room temperature for 1 h. Proteins were visualized using a pierce enhanced chemiluminescence detection kit (Thermo Fisher Scientific), and the intensity of the bands was measured using ImageJ software 1.42q (National Institutes of Health).

### Statistical analysis

Numerical data are presented as the mean ± SEM. To assess the differences in tonic *I*_NMDA_ under DOCA-salt conditions, we performed a hierarchical testing procedure. In the first step, a Shapiro–Wilk test was used to test the null hypothesis that the data distribution was normal with a significance level of 5%. For data with a normal distribution, the statistical significance of comparisons was assessed using either a two-sample *t* test or a one-way ANOVA followed by a post hoc test (e.g., Bonferroni's test). If the null hypothesis was rejected, nonparametric tests were used with Microcal Origin software (RRID: SCR_002815). For all experiments, male rats were used to avoid effects of hormonal changes on the results. Tonic *I*_NMDA_ was categorized into two modalities: either the Mg^2+^-sensitive tonic *I*_NMDA_, measured by *I*_holding_ shift induced by increasing the extracellular Mg^2+^ concentration from 20 μM to 1.2 mM at *V*_h_, −70 mV, or the Mg^2+^-resistant tonic *I*_NMDA_, measured by *I*_holding_ shift induced by NMDAR antagonists at *V*_h_, −70 mV in normal aCSF. Electrophysiological recordings were taken from three or more animals per group, and three to four slices were collected per animal. The Mann–Whitney test was used to compare two groups if the data were not normally distributed. The pharmacological sensitivity of tonic *I*_NMDA_ to various NMDAR antagonists was investigated. To compare *I*_NMDA_ amplitude and protein expression (NR2A, NR2B, and NR2D) in the H_2_O group and DOCA-salt group, we used one-way ANOVA followed by a post hoc test.

## Results

### Transient increase of *I*_NMDA_ in SON MNCs in DOCA-salt rats

**Figure 1.**
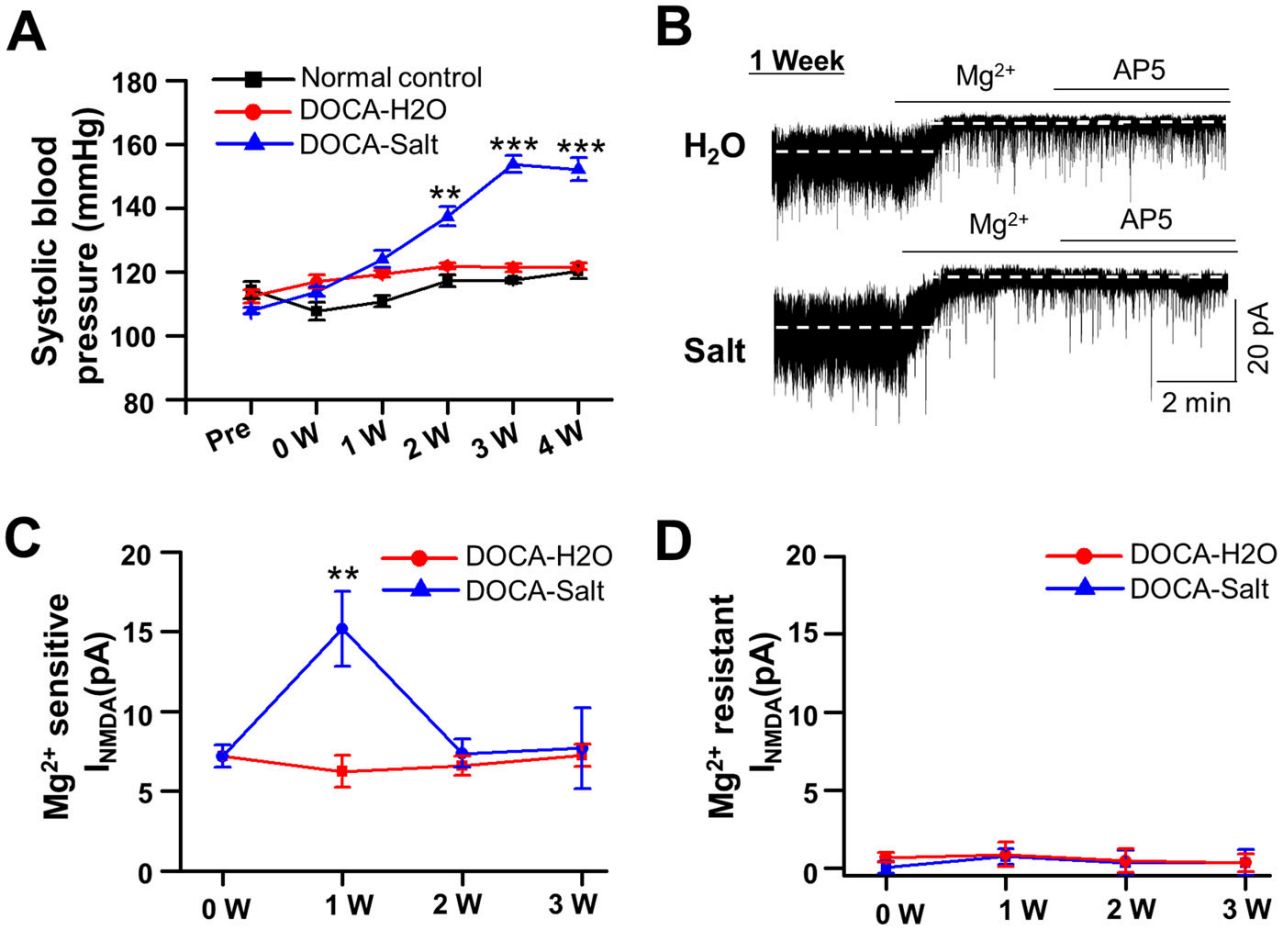
Increased *I*_NMDA_ of SON MNCs in DOCA-salt rats. ***A***, Systolic blood pressure at different times in naive control (normal control, *n* = 6), DOCA-H_2_O (*n* = 6), and DOCA-salt model rats (DOCA-salt, *n* = 18) (*p < 0.05, ***p < 0.001, compared with normal control). ***B***, Representative current traces showing effects of Mg^2+^, followed by the sequential application of AP5 (200 µM), a NMDA receptor antagonist, in holding current in DOCA-H_2_O and DOCA-salt model rats at 1 week. Representative current traces show the effect of sequential application of Mg^2+^ (1.2 mM) and additional AP5 (100 mM) on the holding current of SON MNCs at 1, 2, and 4 weeks. ***C***,***D***, Summarized bar graph showing average tonic current amplitude block by Mg^2+^ (***C***) and additional AP5 (***D***) in each week, respectively. **p < 0.01, compared with DOCA-H_2_O.

DOCA-salt treatment successfully induced hypertension as shown by a time-dependent increase in systolic arterial pressure (SAP) in rats. The nephrectomy/DOCA-salt treatment (DOCA-salt) elicited a significant increase in the SAP (*F*_(2,72)_ = 48.03; *p* = 0.01; two-way ANOVA). SAP tended to increase at 1 week, increased significantly at 2 weeks, reached the maximal hypertension at 3 weeks, and was maintained up to 4 weeks post-DOCA implantation (PDI) in DOCA-salt group, while the nephrectomy/DOCA alone with no NaCl and KCl (DOCA-H_2_O) did not affect SAP during the period compared with normal control animals (*p* = 0.62; Bonferroni’s post hoc test following two-way ANOVA; Fig. 1*A*).

Increased plasma vasopressin (VP), a neurohypophysial hormone, level may affect the fluid homeostasis (Boone and Deen, 2008; Parekh et al., 2021). Although SAP increase did not reach statistical significance at 1 week PDI (DOCA-H_2_O 119.34 ± 0.975 mmHg, *n* = 5 vs DOCA-salt 123.99 ± 2.69 mmHg, *n* = 18 rats), the volume of water intake and urine output were significantly increased in DOCA-salt than DOCA-H_2_O groups at 1 week PDI (Table 1). In addition, the urine osmolality was significantly lower in DOCA-salt than that in DOCA-H_2_O rats, meanwhile the serum osmotic pressure was not different in the two groups (Table 1).

**Table 1.** Changes in metabolic parameters after 1 week in DOCA-salt induced hypertensive model

Parameters	DOCA-H_2_O	DOCA-Salt	*p* value
Serum osmolality (mOsm/L)	313 ± 3.26	310 ± 1.45	0.40
Urine osmolality (mOsm/L)	1,098 ± 60.24	657 ± 31.49	<0.001
Water intake (ml/d)	15 ± 4.97	145 ± 13.67	<0.001
Urine output (ml/d)	13.66 ± 2.58	127 ± 14.56	<0.001

In next experiment, we investigated *I*_NMDA_ in SON MNCs in DOCA-H_2_O and DOCA-salt groups at 1, 2, and 4 weeks PDI. Increasing [Mg^2+^]_o_ from 20 µM to 1.2 mM induced a significantly larger *I*_holding_ in DOCA-salt (15.17 ± 2.32 pA, *n* = 19 neurons from 7 rats) compared with DOCA-H_2_O group (6.25 ± 0.98 pA, *n* = 9 neurons from 3 rats) at 1 week PDI (Fig. 1*B*,*C*), while the difference was not observed at 2 and 4 weeks PDI. An additional NMDAR antagonist, AP5, failed to cause further *I*_holding_ shift in all tested groups (Fig. 1*B*,*C*). These results suggested that more NMDARs could be activated to generate Mg^2+^-sensitive *I*_NMDA_ in SON MNCs in DOCA-salt rats at 1 week PDI.

### Pharmacology of the enhanced *I*_NMDA_ in DOCA-salt rats

**Figure 2.**
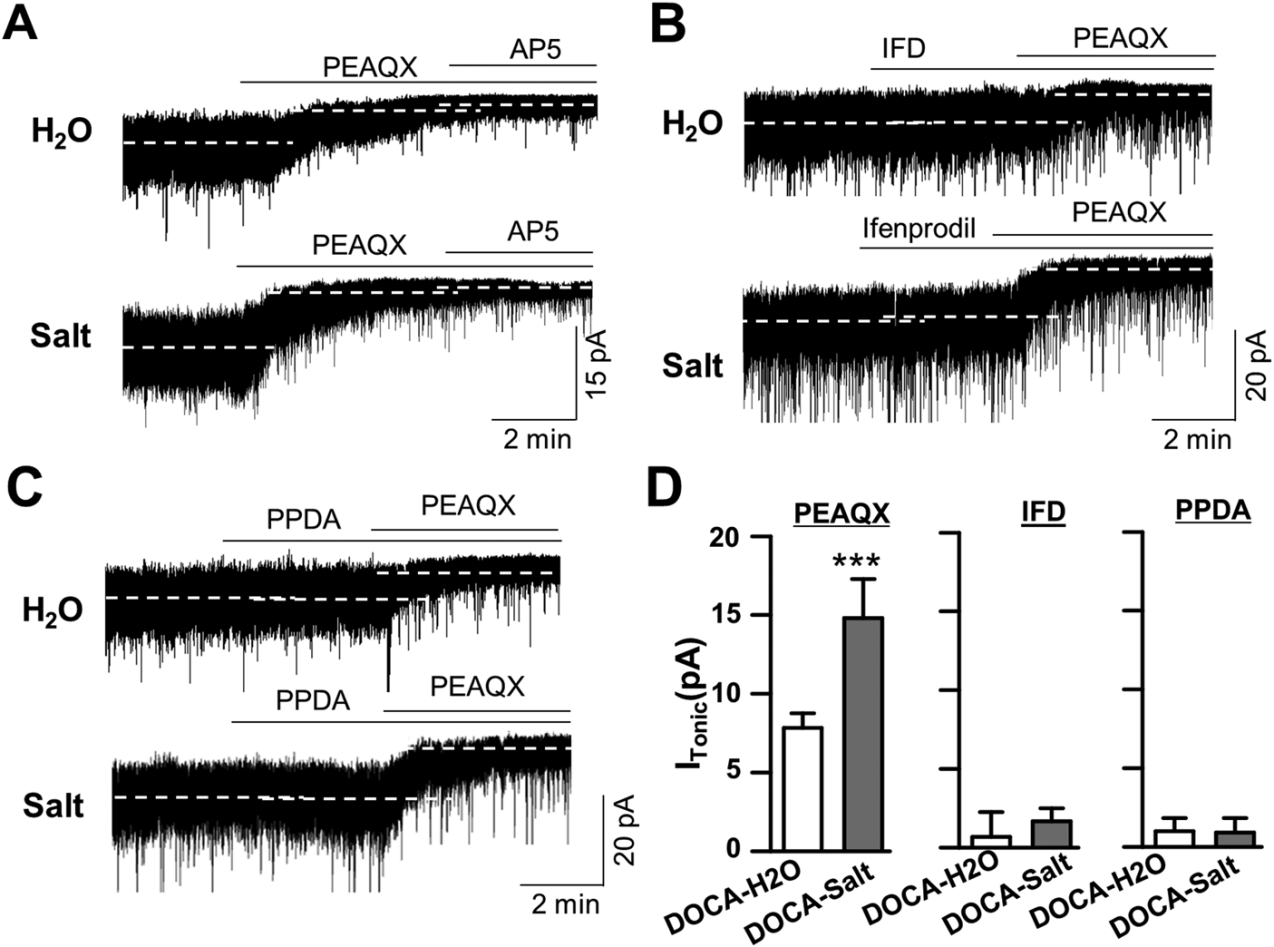
Pharmacology of *I*_NMDA_ in DOCA-H_2_O and DOCA-salt group. ***A–C***, Representative current traces showing the effect of PEAQX (1 µM), a NR2A receptor antagonist (***A***), ifenprodil (30 µM), an NR2B receptor antagonist (***B***), and PPDA (1 µM), an NR2C/D antagonist (***C***) on holding current. Note that ifenprodil and PPDA caused minimal effects on *I*_holding_, while additional PEAQX caused larger *I*_holding_ changes in DOCA-salt groups. ***D***, Summarized bar graph showing mean of holding current change by each antagonist. *p < 0.05, compared with DOCA-H_2_O; *n* = 6 and 8 in DOCA-H_2_O and DOCA-salt, respectively.

To assess the composition of NMDARs that potentiate Mg^2+^-sensitive *I*_NMDA_ at 1 week PDI, we investigated *I*_NMDA_ at *V*_holding_ −70 mV in low Mg^2+^ aCSF and compared its sensitivity with NR2A, NR2B, and NR2C/D subunit selective antagonists in DOCA-H_2_O and DOCA-salt group (Fig. 2).

PEAQX, an NR2A subunit antagonist, uncovered *I*_NMDA_ (*I*_PEAQX_) of nondepolarized SON MNCs under low Mg^2+^ condition in both DOCA-H_2_O group (7.84 ± 0.92 pA; *n* = 6 neurons from 3 rats; *F*_(2,15)_ = 16.23; *p* = 0.003; Bonferroni’s post hoc test following one-way RM-ANOVA) and DOCA-salt (14.80 ± 2.49 pA; *n* = 8 neurons from 3 rats; *F*_(2,15)_ = 33.14; *p* < 0.001; Bonferroni’s post hoc test following one-way RM-ANOVA). *I*_PEAQX_ was significantly larger in DOCA-salt than that in DOCA-H_2_O group (*p* < 0.001, two-sample *t* test). However, additional AP5 (PEAQX + AP5) caused minimal *I*_holding_ shift in both groups (DOCA-H_2_O, 0.80 ± 1.42 pA, vs DOCA-salt, 0.77 ± 0.94 pA; Fig. 2*A*,*D*). In contrast, ifenprodil (IFD, a selective NR2B antagonist) failed to cause *I*_holding_ shift in both DOCA-H_2_O (*F*_(2,15)_ = 9; *n* = 6 neurons from 3 rats; *p* = 0.93; Bonferroni’s post hoc test following one-way RM-ANOVA) and DOCA-salt (*F*_(2,18)_ = 33.22; *n* = 8 neurons from 4 rats; *p* = 0.99; Bonferroni’s post hoc test following one-way RM-ANOVA), while additional PEAQX uncovered *I*_NMDA_ in both groups (DOCA-H_2_O, *p* = 0.01, and DOCA-salt, *p* < 0.001; Bonferroni’s post hoc test following one-way RM-ANOVA in both cases). Notably, additional PEAQX (IFD + PEAQX) uncovered larger *I*_NMDA_ in DOCA-salt compared with DOCA-H_2_O group (DOCA-H_2_O, 6.80 ± 1.73 pA vs DOCA-salt, 14.27 ± 2.17 pA; *p* < 0.001; two-sample *t* test).

Similarly, PPDA (a selective NR2C/2D antagonist) failed to cause *I*_holding_ shift in the both DOCA-H_2_O (*F*_(2,21)_ = 10.99; *n* = 8 neurons from 3 rats; *p* = 0.65; Bonferroni’s post hoc test following one-way RM-ANOVA) and DOCA-salt (*F*_(2,27)_ = 42.56; *n* = 10 neurons from 4 rats; *p* = 0.84; Bonferroni’s post hoc test following one-way RM-ANOVA), while additional PEAQX uncovered *I*_NMDA_ in both groups (DOCA-H_2_O, *p* = 0.009, and DOCA-salt, *p* < 0.001; Bonferroni’s post hoc test following one-way RM-ANOVA in both cases). Consistently, additional PEAQX (PPDA + PEAQX) uncovered larger *I*_NMDA_ in DOCA-salt compared with DOCA-H_2_O group (DOCA-H_2_O, 5.45 ± 1.73 pA vs DOCA-salt, 12.58 ± 1.62 pA; *p* < 0.001; two-sample *t* test). These results suggested that Mg^2+^-sensitive *I*_NMDA_ predominantly represented the activation of NR2A-containing NMDARs in nondepolarized SON MNCs in both DOCA-H_2_O and DOCA-salt groups.

### Decreased EAAT activity in the SON of DOCA-salt rats

**Figure 3.**
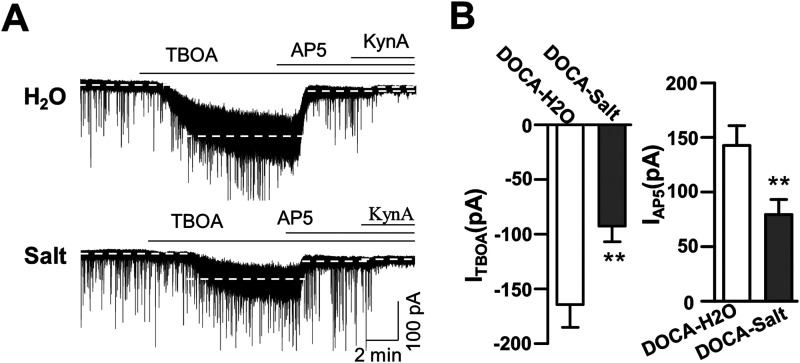
Comparison of *I*_NMDA_ in the presence of EAAT antagonist, TBOA, in DOCA-H_2_O and DOCA-salt models. ***A***, Representative current traces showing the effects of TBOA, a glutamate transporter blocker (100 µM), followed by the sequential application of AP5 (200 µM), an NMDA receptor antagonist and kynurenic acid (5 mM), a glutamate receptor antagonist, in holding current. Note that AP5 completely reversed *I*_holding_ change induced by TBOA in both DOCA-H_2_O and DOCA-salt. ***B***, Summarized bar graph showing the mean of holding current change by TBOA (left) and additional AP5 (right). *p < 0.05, compared with DOCA-H_2_O; *n* = 6 and 7 in DOCA-H_2_O and DOCA-salt, respectively.

EAATs that uptake glutamate, particularly the astrocytic glutamate transporter-1 (GLT1) and glutamate/aspartate transporter (GLAST) isoforms, potently regulate glutamate clearance to maintain ambient glutamate levels in the central nervous system (Fleming et al., 2011; Sun et al., 2014). Increased neurohumoral drive such as heart failure condition-induced glial remodeling caused decreased GLT1 and increased GLAST to elevate ambient glutamate level; thus, increased *I*_NMDA_ of SON NMCs (Potapenko et al., 2012) and similar changes in GLT1 was also observed in SON MNCs during dehydration (Fleming et al., 2011). To know if this is the case in DOCA-salt rats, we compared endogenous EAAT activity by quantifying the magnitude of the *I*_NMDA_ evoked by a EAAT blocker in DOCA-H_2_O and DOCA-salt rats (Fig. 3).

Bath application of the nonselective EAAT blocker TBOA (100 µM) induced a large inward shift in *I*_holding_ (*I*_TBOA_) in SON MNCs in both DOCA-H_2_O (*F*_(2,15)_ = 54.09; *n* = 6 neurons from 3 rats; *p* < 0.001; Bonferroni’s post hoc test following one-way RM-ANOVA) and DOCA-salt (*F*_(2,18)_ = 34.57; *n* = 7 neurons from 3 rats; *p* < 0.001; Bonferroni’s post hoc test following one-way RM-ANOVA). Interestingly, TBOA induced inward shift in *I*_holding_ (*I*_TBOA_) was significantly smaller in DOCA-salt (DOCA-salt, −92.74 ± 14.48 pA) than that in DOCA-H_2_O group (−164.47 ± 20.92 pA; *p* = 0.01; two-sample *t* test). *I*_TBOA_ was mostly blocked by AP5; thus, the additional application of kynurenic acid, KynA (AP5 + KynA), induced only a minimal *I*_holding_ shift in both groups (Fig. 3*A*). These results suggested that attenuated glutamate transporter activity increased extracellular glutamate concentration, resulting in turn in the activation of a Mg^2+^-sensitive *I*_NMDA_ in DOCA-salt group.

### Exogenous glutamate equalizes *I*_NMDA_ and *I*_PEAQX_ in DOCA-H_2_O and DOCA-salt rats

**Figure 4.**
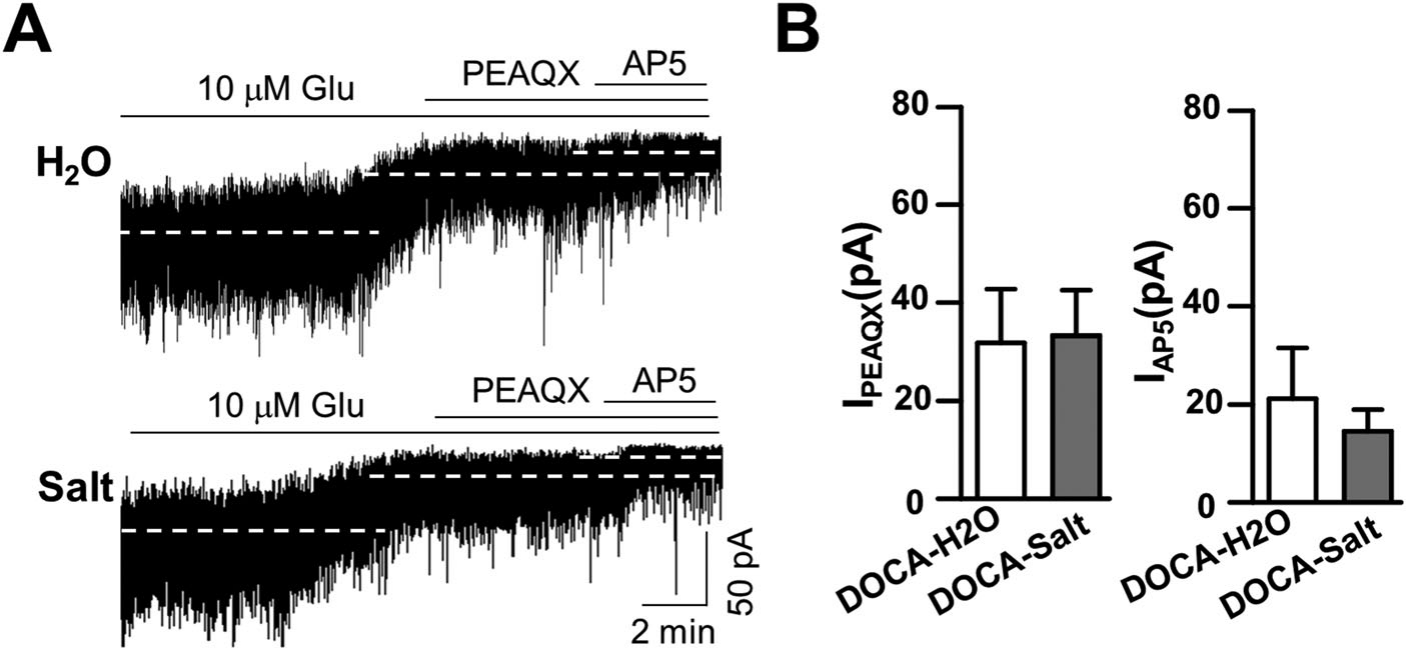
Comparison of *I*_NMDA_ in the presence of exogenous glutamate in DOCA-H_2_O and DOCA-salt groups. ***A***, Representative current traces show the effect of PEAQX and additional AP5 (100 µM) on the holding current of SON MNCs. Note that PEAQX and AP5 caused similar changes in *I*_holding_. ***B***, Summarized bar graph showing mean of holding current change by PEAQX (left) and additional AP5 (right); *n* = 7 and 9 in DOCA-H_2_O and DOCA-salt, respectively.

Next, we further tested whether increasing extracellular glutamate levels potentiated the Mg^2+^-sensitive *I*_NMDA_ in SON MNCs in DOCA-salt group compared with those in DOCA-H_2_O rats (Fig. 4).

In the presence of glutamate (10 µM), both PEAQX and additional AP5 (PPDA + AP5) caused significant changes in both DOCA-H_2_O (*F*_(2,15)_ = 6.09; *p* = 0.05; one-way RM-ANOVA, *n* = 6 neurons from 3 rats) and DOCA-salt (*F*_(2,21)_ = 12.82; *p* < 0.001; one-way RM-ANOVA; *n* = 8 neurons from 3 rats). *I*_PEAQX_ was not different in DOCA-H_2_O (31.87 ± 10.89 pA) from DOCA-salt group (33.25 ± 9.3 pA; *p* = 0.867; two-sample *t* test). Additional AP5 (PEAQX + AP5) induced similar outward shift in *I*_holding_ in both groups (DOCA-H_2_O, 21.16 ± 10.38 pA vs DOCA-salt, 14.47 ± 4.47 pA; two-sample *t* test; *p* = 0.351). As a result, total *I*_NMDA_ was not different in DOCA-H_2_O (53.03 ± 21.14 pA) and DOCA-salt groups (47.73 ± 12.43 pA; two-sample *t* test; *p* = 0.820).

These results suggested that the increased *I*_NMDA_ in DOCA-salt rats was due to blunted GLUT activity, leading to increased levels of endogenous glutamate, but not due to increase or changes in extrasynaptic NMDARs. Moreover, these results indicate that *I*_NMDA_ is mediated by NR2A receptors in both groups.

**Figure 5.**
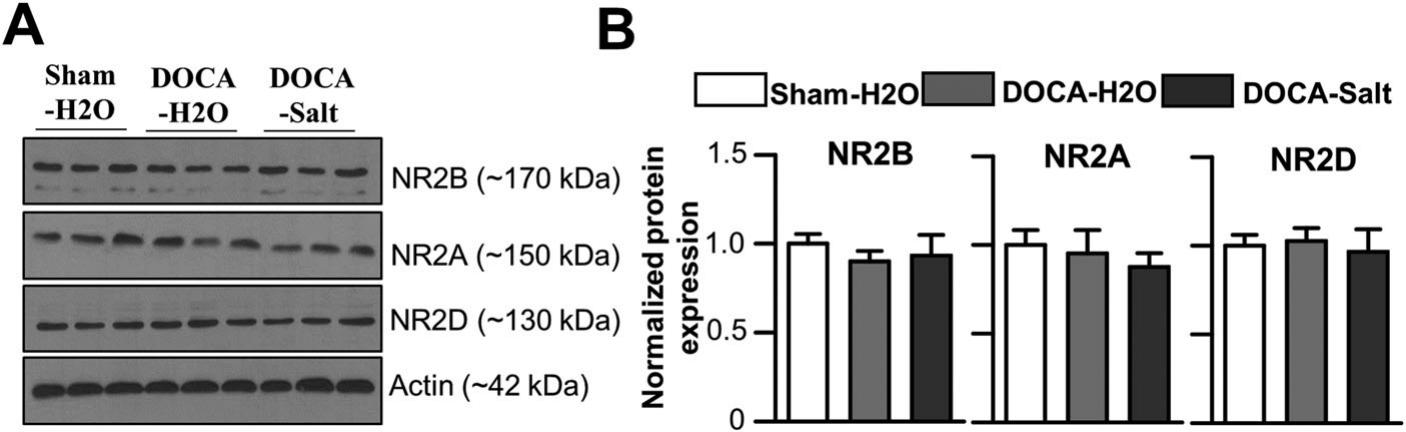
The expression of NMDAR subunit in the DOCA-H_2_O and DOCA-salt groups. ***A***, Representative image of a Western blot showing NR2A, NR2B, and NR2D subunit expression in DOCA-H_2_O and DOCA-salt groups. ***B***, Summarized bar graph showing the relative expression of NMDRAs in the SON of DOCA-H_2_O and DOCA-salt groups. The protein expression was normalized to the level detected in the DOCA-H_2_O group and compared with the expression in DOCA-salt animals. Summarized data shown are the mean ± SE (*n* = 3 rats).

To determine whether changes in the expression of NMDAR subunits contributed to altered *I*_NMDA_ in DOCA-salt rats, we compared the expression of NR2A-D in DOCA-H_2_O and the DOCA-salt groups at 1 week PDI. Western blot results showed no significant difference in NR2A-D protein expression between the groups (Fig. 5*A*,*B*).

### *I*_ifenprodil_ in depolarized SON MNCs did not differ between DOCA-H_2_O and DOCA-salt rats

**Figure 6.**
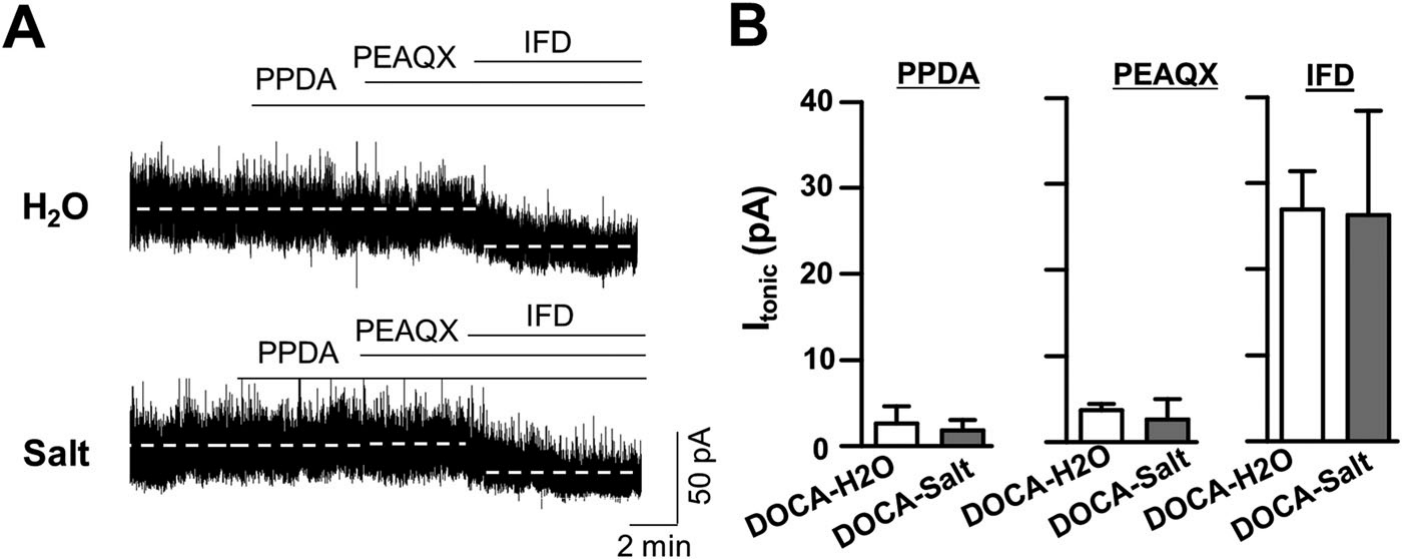
Pharmacology of *I*_NMDA_ in depolarized SON MNCs. ***A***, Representative traces showing the *I*_holding_ changes induced by the sequential addition of 1 µM PPDA, 1 µM PEAQX, and 10 µM IFD in DOCA-H_2_O and DOCA-salt groups with a depolarized membrane potential (Vh, +40 mV). The dotted lines indicate the mean *I*_holding_ under each condition. ***B***, Summarized *I*_holding_ changes induced by PPDA, PEAQX, and IFD in SON MNCs from both DOCA-H_2_O and DOCA-salt groups; *n* = 6 in each group.

We next investigated the sensitivity of *I*_NMDA_ to NR2A, NR2B, and NR2C/D subunit selective antagonists in depolarized neurons (*V*_holding_ +40 mV; Fig. 6).

In agreement with the previous reports (Fleming et al., 2011; Neupane et al., 2021), IFD caused an inward *I*_holding_ shift (*I*_ifenprodil_) in depolarized SON MNCs in both DOCA-H_2_O (*F*_(2,15)_ = 19.25; *n* = 6 neurons from 3 rats; *p* < 0.001; Bonferroni’s post hoc test following one-way RM-ANOVA) and DOCA-salt (*F*_(2,18)_ = 32.16; *n* = 7 neurons from 3 rats; *p* < 0.001; Bonferroni’s post hoc test following one-way RM-ANOVA). *I*_ifenprodil_ was not different in DOCA-H_2_O (26.93 ± 4.46 pA) and DOCA-salt group (26.25 ± 12.74 pA; two-sample *t* test; *p* = 0.960). In contrast, PEAQX and PPDA failed to cause significant *I*_holding_ changes in both DOCA-H_2_O (PEAQX, *p* = 0.96 and PPDA, *p* = 0.91; Bonferroni’s post hoc test following one-way RM-ANOVA) and DOCA-salt rats (PEAQX, *p* = 0.95 and PPDA, *p* = 0.89; Bonferroni’s post hoc test following one-way RM-ANOVA in both cases; Fig. 5*C*,*D*). These results suggested that *I*_ifenprodil_ in depolarized SON MNCs did not contribute to the *I*_NMDA_ mediated by increased ambient glutamate at 1 week PDI.

### *I*_PEAQX_ but not *I*_ifenprodil_ sensed an increased ambient glutamate concentration in DOCA-salt rats

**Figure 7.**
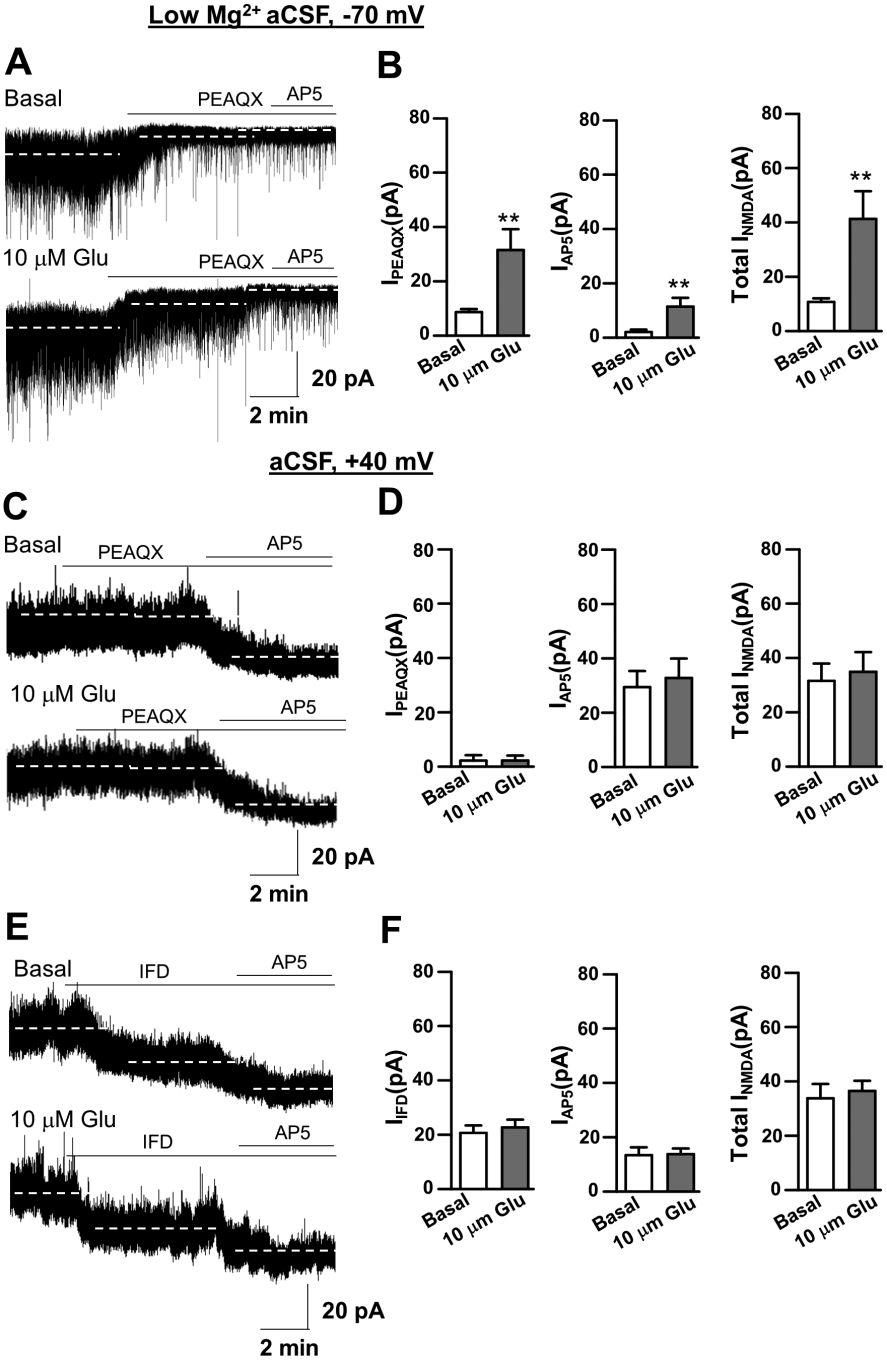
Mechanism of generating *I*_NMDA_ in the presence of exogenous glutamate in nondepolarized and depolarized SON MNCs. ***A***, Representative current traces show the effect of PEAQX and additional AP5 (100 µM) on the holding current of nondepolarized SON MNCs in basal condition and in the presence of 10 µM glutamate. ***B***, Summarized bar graph showing mean of holding current change by PEAQX (left), additional AP5 (middle), and total *I*_NMDA_ (right); *n* = 7 and 9 in basal and in the presence of 10 µM, respectively. ***C***, Representative current traces show the effect of PEAQX and additional AP5 (100 µM) on the holding current of depolarized SON MNCs in basal condition and in the presence of 10 µM glutamate. ***D***, Summarized bar graph showing mean of holding current change by PEAQX (left), additional AP5 (middle), and total *I*_NMDA_ (right); *n* = 6 in both condition. ***E***, Representative current traces show the effect of ifenprodil and additional AP5 (100 µM) on the holding current of depolarized SON MNCs in basal condition and in the presence of 10 µM glutamate. ***F***, Summarized bar graph showing mean of holding current change by IFD (left), additional AP5 (middle), and total *I*_NMDA_ (right); *n* = 7 and 6 in basal and in the presence of 10 µM, respectively.

In the next experiments, we directly investigated the hypothesis that altered ambient glutamate concentration could be sensed by *I*_PEAQX_ in nondepolarized SON MNCs. For this, we compared the effects of PEAQX on *I*_NMDA_ in the absence and presence of exogenous glutamate (10 µM; Fig. 7).

As expected, exogenous glutamate significantly increased *I*_PEAQX_ in nondepolarized SON MNCs from 8.67 ± 1.10 pA (*n* = 7 neurons from 3 rats) to 31.49 ± 7.71 pA (*n* = 6 neurons from 3 rats). Additional AP5 (PEAQX + AP5) caused a minimal but significant *I*_holding_ shift in the presence of exogenous glutamate (control, 2.06 ± 0.85 pA, *n* = 7 neurons from 3 rats, vs 10 µM glutamate, 11.37 ± 3.24 pA, *n* = 6 neurons from 3 rats; *p* = 0.01 in both cases; two sample *t* test). As a result, total *I*_NMDA_ in the nondepolarized condition was significantly larger in the presence of 10 µM glutamate (10.73 ± 1.35 pA; *n* = 6 neurons from 3 rats) compared with the control condition (41.24 ± 10.25 pA, *n* = 7 neurons from 3 rats; Fig. 7*B*; *p* = 0.01; two-sample *t* test), while the portion of *I*_PEAQX_ to the total *I*_NMDA_ was not different in the absence and presence of glutamate (82 ± 6.67%, *n* = 6 vs 76 ± 5.49%, *n* = 7; *p* = 0.50; two-sample *t* test). Noted that IFD did not affect *I*_holding_ in nondepolarized SON MNCs even in the presence of exogenous glutamate (control, 1.67 ± 0.95 vs 10 µM glutamate, 1.25 ± 1.21 pA). These results supported the idea that *I*_NMDA_ predominantly represented the activation of NR2A-containing NMDARs in nondepolarized SON MNCs.

We further investigated whether the increased ambient glutamate concentration could be sensed by *I*_PEAQX_ and *I*_ifenprodil_ in depolarized SON MNCs. In contrast to *I*_PEAQX_ in nondepolarized neurons, exogenous glutamate failed to increase *I*_PEAQX_ of depolarized SON MNCs (control, 2.18 ± 1.98 pA, *n* = 6 neurons from 3 rats; *F*_(2,15)_ = 24.59; *p* = 0.90; Bonferroni’s post hoc test following one-way RM-ANOVA and 10 µM glutamate, 2.20 ± 1.84 pA, *n* = 6 neurons from 3 rats; *F*_(2,15)_ = 20.97; *p* = 0.92; Bonferroni’s post hoc test following one-way RM-ANOVA). Additional AP5 (PEAQX + AP5) uncovered a similar *I*_NMDA_ in both conditions (control, 29.39 ± 5.92 pA and 10 µM glutamate, 6.67 ± 7.25 pA; Fig. 7*C,D*). As a result, the total *I*_NMDA_ of depolarized SON MNCs was not different in the absence and presence of exogenous glutamate (Fig. 7*C*,*D*).

In addition, although IFD caused an inward *I*_holding_ shift in depolarized SON MNCs (Figs. 6, 7*C*,*D*), exogenous glutamate failed to affect *I*_ifenprodil_ in depolarized SON MNCs (control, 20.38 ± 2.68 pA, *n* = 7 neurons from 3 rats vs glutamate, 22 ± 2.90 pA, *n* = 6 neurons from 3 rats). Additional AP5 (IFD + AP5) uncovered a similar magnitude of *I*_NMDA_ in both conditions (Fig. 7*E*,*F*). As a result, total *I*_NMDA_ in depolarized SON MNCs was not different in the absence and presence of exogenous glutamate (Fig. 7*E*,*F*) suggesting that membrane depolarization to *V*_holding_ of +40 mV maximized *I*_NMDA_ in our recording conditions, thus making it insensible to increased ambient glutamate at 1 week PDI.

## Discussion

The main findings of this study are that (1) Mg^2+^-sensitive *I*_NMDA_ was increased significantly but transiently at post 1 week in DOCA-salt rats; (2) the enhanced *I*_PEAQX_ is an agreement with attenuated EAAT activity with no changes in NMDAR subunit expression at 1 week PDI; and (3) *I*_ifenprodil_ in depolarized SON MNCs did not respond to increased ambient glutamate in normal and DOCA-salt groups. These findings indicate that the *I*_NMDA_ in nondepolarized and depolarized SON MNCs is dominantly mediated by NR2A- and NR2B-containing NMDARs, respectively, and the former efficiently sensed the increased ambient glutamate concentration in the SON of normal and hypertensive rats. One limitation of this study is that only male rats were used to avoid the potential hormonal changes in female rats, which directly influence the hypertension pathophysiology. To the best of our knowledge, this is the first evidence that NR2A-containing NMDARs could contribute to tonic excitation mediated by extrasynaptic NMDARs.

### *I*_NMDA_ generated by NR2A-containing NMDARs

Glutamate can generate a tonic *I*_NMDA_ when it binds to eNMDARs, while it evokes classical EPSCs via the activation of their synaptic counterparts. Tonic NMDAR current, *I*_NMDA_, is a hallmark of eNMDAR activity, and NR2A subunit expression is more localized at synaptic sites and exclusively observed in postsynaptic sites (Groc et al., 2006; Paoletti et al., 2013; Cercato et al., 2016; Franchini et al., 2020). Although the presence of NR2A in both sites has been reported (Thomas et al., 2006; Gladding and Raymond, 2011), it was surprising to observe that PEAQX, an NR2A subunit antagonist, blocked *I*_NMDA_ in the present study (Fig. 2). These results suggest that synaptic NR2A-containing NMDARs rather than eNMDARs containing NR2B subunit could generate the Mg^2+^-sensitive *I*_NMDA_ in nondepolarized SON MNCs. The idea is supported by our results that eNMDAR antagonists including ifenprodil and PPDA failed to affect *I*_holding_ of nondepolarized SON MNCs in low Mg^2+^ condition (Fig. 2). It is noteworthy that the tonic activation of GABA_A_ receptors generating tonic GABA_A_ inhibition were identified superimposed to high-frequency synaptic events (Otis et al., 1991; Salin and Prince, 1996; Hausser and Clark, 1997). Thus, *I*_PEAQX_ could represent the superimposition of high-frequency synaptic NMDAR currents especially in nondepolarized neurons under low [Mg^2+^]_o_ conditions. The idea is also in line with the fact that Mg^2+^ enhances the desensitization of NMDARs (Kampa et al., 2004), while the kinetics of NMDARs are faster at more negative holding potentials (Konnerth et al., 1990; Keller et al., 1991).

Combined with their extrasynaptic location (Lozovaya et al., 2004), NR2B-containing eNMDARs have been known to generate *I*_NMDA_ when exposed to low concentration of ambient glutamate. However, ifenprodil failed to uncover *I*_NMDA_ in nondepolarized SON MNCs even in the presence of exogenous glutamate (Figs. 2, 7). The apparent discrepancy may be reconciled with the idea that Mg^2+^ unblock, coupled with membrane potential depolarization, is essential to activate NR2B-containing receptors in our recording condition. This idea is in agreement with the fact that ifenprodil uncovered *I*_NMDA_ in depolarized SON MNCs (Figs. 6, 7). Given that NR2A and NR2B confer similar glutamate sensitivity to NMDARs (Erreger et al., 2007; Hansen et al., 2008) and low [Mg^2+^]_o_ (20 µM) efficiently blocked NMDARs in nondepolarized SON MNCs, these results are also supportive of the idea that EPSCs causing a transient increase of glutamate concentration in the synaptic cleft activates synaptic NR2A receptors to generate *I*_PEAQX_ in nondepolarized SON MNCs.

However, combined with the fact that EPSC frequency was not different in the DOCA-H_2_O and DOCA-salt groups (Table 2), the significantly larger *I*_PEAQX_ in DOCA-salt rats argues against a contribution of superimposed EPSCs mediating *I*_PEAQX_. This is further supported by our results showing that increased ambient glutamate equalized *I*_PEAQX_ in the DOCA-salt and DOCA-H_2_O groups (Fig. 4). Thus, it is noteworthy that peri- as well as extrasynaptic receptors contribute tonic NMDAR currents (Papouin and Oliet, 2014) as GABA_A_ tonic inhibition (Farrant and Nusser, 2005; Glykys and Mody, 2007; Belelli et al., 2009). Future studies are warranted to investigate whether perisynaptic NMDARs containing NR2A subunit could generate PEAQX-sensitive *I*_NMDA_ in low [Mg^2+^]_o_ condition and, thus, also respond to an increased ambient glutamate concentration such as in DOCA-salt 1 week.

**Table 2.** Phasic current properties of SON MNCs

EPSC properties	DOCA-H_2_O	DOCA-salt	*p* value
Frequency (Hz)	0.99 ± 0.33	0.88 ± 0.20	0.76
Amplitude (pA)	43.02 ± 3.74	40.46 ± 1.95	0.51
Weighted *τ* (ms)	3.49 ± 0.65	3.28 ± 0.34	0.75

### *I*_PEAQX_ but not by *I*_ifenprodil_ contribute to sensing altered ambient glutamate concentrations in DOCA-salt rats

In the present study, NR2A-containing NMDARs generated *I*_PEAQX_ in nondepolarized SON MNCs in low [Mg^2+^]_o_ condition, which sensed an increased ambient glutamate, thus generating in turn a larger *I*_NMDA_ in DOCA-salt rats. In contrast, *I*_ifenprodil_ in depolarized SON MNCs was not different in DOCA-H_2_O and DOCA-salt groups (Fig. 6). One possible explanation for this difference is that *I*_ifenprodil_ in depolarized SON MNCs was saturated in our recording condition. This possibility is actually supported by our results showing that exogenous glutamate failed to increase *I*_ifenprodil_ in depolarized SON MNCs (Fig. 7). This result is also in line with the fact that *I*_ifenprodil_ was not potentiated following an increase in ambient glutamate concentration in DOCA-salt rats (Fig. 2).

NMDAR phosphorylation reduces the voltage-dependent Mg^2+^ block of the channels (Chen and Huang, 1992), leading to, as recently shown (Pham et al., 2022), an increase in *I*_ifenprodil_. The lack of increase in *I*_ifenprodil_ in this study suggests that NR2B phosphorylation is not a likely mechanism contributing to changes in NMDAR function reported in DOCA-salt rats. Overall, our results showed that *I*_ifenprodil_ represents the saturated activity of NR2B-containing NMDARs in depolarized SON MNCs.

Although it is purely speculative, it is interesting to note that “the extrasynaptic space” comprising separate domains was proposed instead of one large homogeneous volume (Papouin and Oliet, 2014). Given that NR2A and NR2B are preferentially located in synaptic and extrasynaptic regions (Kew et al., 1998; Tovar and Westbrook, 1999; Barria and Malinow, 2002), *I*_PEAQX_ and I_Ifenprodil_ may preferentially sense glutamate concentrations in the synaptic cleft over the ambient glutamate concentration in extrasynaptic space, respectively. However, our results showing that exogenous glutamate increased *I*_PEAQX_ argue against the notion of a functional barrier discriminating “PEAQX-sensitive” synaptic NMDARs and “ifenprodil-sensitive” extrasynaptic regions. Future studies are warranted to identify the spatial component differentiating “PEAQX-sensitive regions” over “ifenprodil-sensitive regions” in the SON.

### An increase in ambient glutamate was insufficient to generate a Mg^2+^-resistant *I*_NMDA_

In a previous report (Neupane et al., 2021), it was shown that eNMDARs containing homodimeric NR2D subunit were the best candidate generating the Mg^2+^-resistant *I*_NMDA_ in both nondepolarized and depolarized SON MNCs. However, an “NR2D recall” is essential to generate the Mg^2+^-resistant *I*_NMDA_ in the matured brain, because NR2D expression is gradually decreased with brain maturation (Monyer et al., 1994; Dunah et al., 1996; Wenzel et al., 1996; Liu and Wong-Riley, 2010). In the present study, DOCA-salt failed to generate an Mg^2+^-resistant *I*_NMDA_ in SON MNCs, despite the fact that an increase in ambient glutamate concentration was observed at 1 week PDI. Combined with the results that DOCA-salt did not affect NR2D subunit expression (Fig. 5), these results strengthened the idea that “NR2D recall” is essential to generate Mg^2+^-resistant *I*_NMDA_ in the matured brain (Neupane et al., 2021).

### Physiological significance of increased glutamate in SON MNCs in hypertension

Elevated ambient glutamate due to diminished glutamate clearance modifies the excitatory tone that plays a critical role in regulating hypothalamic neurohumoral activation (Potapenko et al., 2012). In hypertension, increased NMDAR-Ca^2+^ responses upregulate neuronal firing in SON MNCs (Zhang et al., 2017; Zhang and Stern, 2017). In the present study, elevated glutamate in the prodromal stage of hypertension was detected by NR2A subunit-containing NMDARs. Given that *I*_NMDA_ mediated by NR2A-containing NMDARs detected only in the physiological atypical concentration of Mg^2+^, it may not directly affect neuronal firing in the SON MNCs. However, normal resting potential appears to be poised in the region of maximal sensitivity to small changes in ambient glutamate and that NR2B subunit-containing NMDARs-mediated tonic *I*_NMDA_ is limited and saturated at depolarized SON MNCs (Neupane et al., 2021; Fig. 7), our results showed that *I*_NMDA_ mediated by NR2A-containing NMDARs is an efficient biosensor for detecting altered ambient glutamate level in the brain.

Taken together, our results suggest that synaptic NMDARs containing NR2A subunit generate *I*_NMDA_ in nondepolarized SON MNCs under low Mg^2+^ condition, while NR2B-containing receptors mediate *I*_NMDA_ in depolarized SON MNCs under normal Mg^2+^ condition. Thus, *I*_PEAQX_ represents the tonic activation of NR2A-containing receptors in SON MNCs, standing thus as a useful biomarker for the detection of ambient glutamate concentration in the SON during normal and pathological neurohumoral overdrive.

## Synthesis

Reviewing Editor: Niraj Desai, National Institute of Neurological Disorders and Stroke Decisions are customarily a result of the Reviewing Editor and the peer reviewers coming together and discussing their recommendations until a consensus is reached. When revisions are invited, a fact-based synthesis statement explaining their decision and outlining what is needed to prepare a revision will be listed below. The following reviewer(s) agreed to reveal their identity: Chris Dulla, Wei Wen.

The manuscript has now been read by two expert reviewers. We all agree that the study has considerable merit and might constitute a valuable addition to the literature. Still, both reviewers had significant questions and concerns. Their reviews are appended below. When you resubmit, please refer to these reviews point by point.

REVIEWER 1

In this manuscript, "Tonic NMDAR currents of NR2A-containing NMDARs represent altered ambient glutamate concentration in the supraoptic nucleus" the authors explore how the tonic extrasynaptic NMDAR current (INMDA) mediated by NR2A-containing NMDARs detected changes in ambient glutamate levels in the supraoptic nucleus (SON). The researchers measured the Mg2+ sensitive INMDA of magnocellular neurosecretory cells (MNCs) at a holding voltage of -70 mV in a low concentration of extracellular magnesium ([Mg2+]o). After inducing a DOCA-salt hypertensive model in rats, they observed a transient but significant increase in INMDA one week later. The increase in INMDA observed with Mg2+ was consistent with the effects induced by a NR2A-selective antagonist, IPEAQX, in both the DOCA-H2O and DOCA-salt groups. Interestingly, the NR2B antagonist, ifenprodil, and the NR2C/D antagonist, PPDA, did not affect the holding current at -70 mV. Further investigations revealed that increased ambient glutamate levels, caused by exogenous glutamate or inhibition of the glutamate transporters, had a smaller effect on NMDA currents in DOCA-salt groups. In contrast, only ifenprodil, but not IPEAQX and PPDA, revealed INMDA at a holding voltage of +40 mV under conditions of 1 mM [Mg2+]o. Importantly, the effect of ifenprodil was not different between the DOCA-H2O and DOCA-salt groups, indicating that NR2B-containing NMDARs are not affected by the hypertensive model. Finally, the researchers examined the protein expression of NR2A, NR2B, and NR2D in the SON and found no significant differences between the DOCA-H2O and DOCA-salt groups. This suggests that the observed changes in glutamate sensing are not due to alterations in the expression levels of these NMDAR subunits. Overall, this study is very rigorous and high quality and is performed by a group with significant expertise in the field. The data are clear and well analyzed. There are a number of moderate concerns that make the data and its impact hard to interpret. The model the authors propose to explain the reason why NR2A, not NR2B, mediate tonic currents is confusing and needs clarification. In addition, the data suggesting that exogenous glutamate application does not increase NR2A currents is hard to reconcile with our understanding of glutamatergic neurotransmission. While this is clearly a strong and high quantity data set, understanding its impact is not so straight forward and requires further clarification.

Major comments:

- What is the relevance of the increased glutamate current if it must be in low Mg2+ to be unmasked at NR2A receptors and does not affect NR2B or NR2D receptors? When would this current occur physiologically? This calls into question the main finding that NR2A sense ambient glutamate because this would only occur in a Mg2+ free/low situation which likely never happens.

- Why do the authors suspect that the change in NR2A tonic current were transient for only 1 week on DOCA-Salt animals?

- Do n values refer to mice or cells? If the later, how many mice per group were studied?

- How do the authors explain a difference in the NR2A (non-depolarized) and NR2B (depolarized) tonic currents in the two animal strains? If DOCA Salt causes a decrease in EAAT function/expression it should raise extracellular glutamate which should affect both NR2A and NR2B signals unless there is some interesting spatial component to the effect. The authors don't explore this sufficiently to interpret this finding.

- The authors state that there is an increase in ambient glutamate 1 weeks after DOCA salt treatment, but their data only shows this indirectly. They should be more precise in describing their conclusion based on the data presented.

- Only male rats were used in this study. Including both male and female rats in this study is important to capture potential sex-specific differences as biological responses and sensitivities can vary between males and females. Hypertension, or high blood pressure, affects both males and females, but the prevalence and patterns can vary between the sexes. This it should be noted and described in the discussion.

Minor comments:

- The labeling of Fig 3A, salt uses a strange font that should be fixed.

- Please avoid using GLUT to refer to glutamate transporters. They are normally referred to as EAATs (excitatory amino acid transporters). GLUT is not the standard term.

- In the text, previous research on GLT1 and GLAST expression in hypertension is described as shifted. Does that mean they are distributed differently? Upregulated? Down regulated? Please clarify.

REVIEWER 2

In this study, the authors investigated the roles of different NMDAR subunits in the modulation of the tonic currents mediated by extrasynaptic NMDARs in the supraoptic nucleus, particularly under the hypertensive condition (DOCA-salt rat model). After showing that the Mg-sensitive tonic current in non-depolarized cells appears to be enhanced in hypertensive animals one week after the DOCA treatment, they go on to demonstrate that this change is mediated by NR2A-containing NMDARs. They further show that this elevated tonic current is caused by reduced activity of glutamate transporters rather than changes in receptor abundance. On the other hand, this tonic current is mediated by NR2B-containing NMDARs in depolarized cells, which does not differ between the control and hypertensive animals. Finally, they show that increasing ambient glutamate concentration via exogenous glutamate application only affects NR2A-mediated tonic currents in non-depolarized cell but not the NR2B-mediated ones in depolarized cells, and conclude that NR2A-containing NMDARs can sense changes in the ambient glutamate concentration in the supraoptic nucleus of hypertensive animals.

Before getting into the scientific soundness and data quality of this study, I would like to point out that the author should make more effort in improving the readability of this manuscript. First, the usage of abbreviations is not consistent; some of them are not annotated properly the first time the corresponding term shows up, and other times the full name of a term is still being used even though the abbreviation has already been established. Second, I find that the meaning of sentences/phrases is ambiguous at multiple places, and some of these should be carefully rephrased as they are essential for the reader's interpretation of the data (see below).

While the observation that NR2A subunit mediates tonic NMDAR currents is interesting, it is perhaps not so surprising as it has been found at extrasynaptic regions before (Gladding and Raymond 2011). Beyond this, the only novel finding is that this NR2A-mediated increase appears to be caused by the hypertensive condition. The authors have not investigated further into the mechanism behind this abnormality other than the TBOA experiment (which has not been tested in the presence of the NR2A blocker, but rather a broad-spectrum blocker APV), and the investigation of NR2B-mediated tonic current in the depolarized neurons feels incomplete (see the 4th concern below). Therefore, I find the contribution of this study to the field in its current form is quite limited.

Concerns:

1. In Fig. 1A, it looks like the blood pressure of the DOCA-H2O group is higher than the normal control group, could the authors provide all test results? In Fig. 1B, the legend states "INMDA at 1, 2, and 4 weeks" whereas the figure only shows the data at 1 week. In Fig. 1C, statistics not shown in the plot. In Fig. 1D, no blue triangle in the plot as labeled in the legend. Furthermore, since the hypertension phenotype is not present at the 1-week timepoint, yet this is the only timepoint that these neurons exhibit an increase in INMDA, could the authors explain why this increase is related to hypertension at all?

In the text (173-195), the authors claim that this is a transient increase, but they have not shown the INMDA measurements before the DOCA implant (fig. 1C). Could the authors include those?

2. From my understanding, in Fig. 2, the authors intend to show that NR2A accounts for the majority of the INMDA by showing that blocking NR2B-D do not lead to significant changes in Iholding, but subsequent blockade of NR2A does. In this case, they should perform multigroup comparison on Itonic measured at the baseline, following NR2B-D blockade, and following NR2A blockade. By the way, Itonic has not been defined either in the figure or the text.

3. In Fig. 3, similarly, the authors should perform multi-group comparison for measurements at baseline, after TBOA treatment, and after APV treatment if they want to argue that TBOA-induced currents are mediated by NMDARs and are different between H2O and salt groups. Perhaps it would make their argument more compelling if they could show whether this change in EAAT activity comes from altered transporter abundance or other sources.

4. In Fig. 6, the authors show that NR2B-mediated INMDA in depolarized neurons does not differ between the two groups, and they explain in discussion that this could be due to the saturation of their measurement, rather than the inability of NR2B to sense this hypertensive state. If this is the case, then it implies that NR2B could also be a sensor just as they claim NR2A to be. Could the authors elaborate on why their measurement could have saturated, and whether there are ways to avoid this caveat?

5. In discussion, the authors interpret their results as "synaptic NR2A-containing NMDARs rather than eNMDARs could generate the Mg2+-sensitive INMDA in non-depolarized SON MNCs", while they have not provide any data indicating that NR2A in these neurons are purely synaptic.

6. In line 335-336, the authors discuss EPSC frequency, but the measurement of this parameter is not described in the Methods.

Sentences/phrases that need clarification (there could be more):

1. Line 11- terms for the two groups not defined.

2. Throughout the abstract, terms such as Iholding and Vholding need to be defined or rephrased.

3. Line 62-63, meaning unclear.

4. Line 129, abbreviation for APV.

5. Line 161, the authors have only mentioned the usage of mice here, if so this information should be provided along with the description of animal models.

6. Line 162-165, meaning unclear.

7. Line 176, the difference shown in the plot is hardly "small".

8. Line 181-187, meaning unclear.

9. Line 214, not all EAATs are glutamate transporters.

10. Line 226, "KynA" not defined.

11. Line 259, meaning unclear, how can currents detect glutamate changes?

12. Line 295-296, not a sentence.

Statistics: The description of statistics in the Methods section does not provide the specific type of non-parametric test used when data is not normally distributed, and no relevant information can be found in the main text or figure legends. The authors should provide the names and all results of the specific test performed for each plot, and pay extra attention to annotate the symbols they use in the figures. Places where improper statistics are performed are listed in the comments to the authors.
